# Real-Time Automatic Segmentation of Optical Coherence Tomography Volume Data of the Macular Region

**DOI:** 10.1371/journal.pone.0133908

**Published:** 2015-08-10

**Authors:** Jing Tian, Boglárka Varga, Gábor Márk Somfai, Wen-Hsiang Lee, William E. Smiddy, Delia Cabrera DeBuc

**Affiliations:** 1 Bascom Palmer Eye Institute, University of Miami, Miami, Florida, United States of America; 2 Department of Ophthalmology, Semmelweis University, Budapest, Hungary; Stanford University Medical Center, UNITED STATES

## Abstract

Optical coherence tomography (OCT) is a high speed, high resolution and non-invasive imaging modality that enables the capturing of the 3D structure of the retina. The fast and automatic analysis of 3D volume OCT data is crucial taking into account the increased amount of patient-specific 3D imaging data. In this work, we have developed an automatic algorithm, OCTRIMA 3D (OCT Retinal IMage Analysis 3D), that could segment OCT volume data in the macular region fast and accurately. The proposed method is implemented using the shortest-path based graph search, which detects the retinal boundaries by searching the shortest-path between two end nodes using Dijkstra’s algorithm. Additional techniques, such as inter-frame flattening, inter-frame search region refinement, masking and biasing were introduced to exploit the spatial dependency between adjacent frames for the reduction of the processing time. Our segmentation algorithm was evaluated by comparing with the manual labelings and three state of the art graph-based segmentation methods. The processing time for the whole OCT volume of 496×644×51 voxels (captured by Spectralis SD-OCT) was 26.15 seconds which is at least a 2-8-fold increase in speed compared to other, similar reference algorithms used in the comparisons. The average unsigned error was about 1 pixel (∼ 4 microns), which was also lower compared to the reference algorithms. We believe that OCTRIMA 3D is a leap forward towards achieving reliable, real-time analysis of 3D OCT retinal data.

## Introduction

Real-time processing and quantitative analysis of retinal images, which has always been of great interest to clinicians, is highly desirable. Quantitative image analysis of the retinal tissue is widely used in the diagnosis and early detection of major blinding diseases, such as glaucoma and age related macular degeneration [1, 2]. Furthermore, many systemic diseases, such as diabetes, can be monitored through the vasculature of the retina [3]. In the last decade, optical coherence tomography (OCT) has emerged as a powerful imaging modality that could provide high-resolution and high-speed cross-sectional images of the retina non-invasively [4]. Recent advancements in OCT imaging have facilitated to capture 3D retinal structures in a few seconds with an axial resolution of ∼ 2 microns [5]. The analysis of OCT volume data requires extensive processing which represents a real challenge nowadays. Taking into account the increased amount of patient-specific imaging data in 3D environments, computational complexity is one of the important factors, which have to be taken into account when considering the number of operations needed to process each 3D dataset. Quality evaluation is not the only criterion to evaluate and compare segmentation methods if we are concerned with real-time (or near real-time) processing. Particularly, in case of real-time segmentation, computational complexity is one of the criteria which has to be carefully considered. The key is to implement an algorithm that can give satisfying segmentation results with low computation load. This is a demandingly needed solution in front of the increased development of cloud technologies and big data analysis, which could impact clinical decision-making.

Segmentation of the retinal layers is one of the first steps to interpret the volumetric OCT data. In general, commercial OCT systems are equipped with proprietary software with limited capabilities for automation and full segmentation of the various cellular layers of the retinal tissue [6]. Many important quantitative features, such as the thickness of the outer nuclear layers, remain unexploited due to the lack of a full retinal segmentation algorithm in most of the commercial devices. Manual segmentation is used to obtain the primary research data in many studies. However, such input from the human graders is time consuming and suffers from inter-observer/intra-observer errors and hence is not suitable for large-scale studies.

The development of an automatic segmentation algorithm for OCT volume data is challenging due to the presence of heavy noise, blood vessels and various pathologies. Unfortunately, there is no single method that could work equally well for segmentation tasks. According to the dimensionality of the input features, we roughly categorized the methods into A-scan based, B-scan based and volume based. Below is a brief overview of the published work related to automatic segmentation of OCT data. A more comprehensive review could be found in [7].

- **A-scan based methods**
  A-scan based methods identify the boundary locations as the intensity peaks or valleys in each A-scan and link the feature points to form a smooth and continuous boundary using different models [8–12]. Advanced denoising methods were usually required and the performance of the algorithms were not robust in all of the images [8–10, 12]. Recent development by Fabritius et al. detected the ILM and RPE in the volume within 17 and 21 seconds respectively [11] with an error less than 5 pixels in 99.7% of the scans.
- **B-scan based methods**
  Commonly seen image segmentation methods, such as thresholding [13], active contour [14], pattern recognition [15, 16] and shortest-path based graph search [17], were also used to detect the boundaries in the B-scans from volume OCT data. The thresholding method proposed by Boyer et al. [13] relied on the absolute value of the intensity, hence the performance of the solution was case dependent and not applicable to other OCT devices. Active contour was customized to detect retinal layers in 20 rodent OCT images by Yazdanpanah el al. [14] and is yet to prove its clinical usefulness in human retina. Pattern recognition based approach introduced by Fuller el al. [15] took advantage of support vector machine (SVM) to estimate the retinal thickness in healthy subjects and patients with macular degeneration, but the accuracy of the detection was low (6 pixels) and the processing time was 10 minutes in training and 2 minutes in running. A random forest classifier approach was employed to estimate the position of retinal layer boundaries with an accuracy of 4.3 microns in 9 boundaries [16]. A graph-based automatic algorithm that could segment eight retinal layers with a thickness error of about 1 pixel has also proven to be robust in images with pathologies [17].
- **Volume based methods**
  Volume scan of the retina is now commonly available in the commercial OCT devices. Recent algorithms exploited the spatial dependency in the adjacent frame to detect retinal surface from 3D data. For example, Garvin et al. [18] and Dufour et al. [19] detected various retinal surfaces from 3D OCT volume by finding the minimum cost feasible surface in the constructed graph as proposed by Li et al. [20]. Two stand-alone softwares developed by Garvin et al. [21] and Dufour et al. [22] are free for research use and are refered to as IOWA Reference Algorithm and Dufour’s software in the rest of the paper.

In this paper, a fast and accurate automatic algorithm that could segment 3D macular OCT data is presented and is refered to as “OCTRIMA 3D”. The acronym OCTRIMA has been previously used to label *OCT retinal image analysis* developed by our group [12] using a different formulation for time domain OCT. Besides aiming for high accuracy and robustness, we have also greatly reduced the processing time to improve the clinical usefulness. The OCTRIMA 3D is a B-scan based method using the shortest-path based graph search framework proposed by Chiu et al. [17] and is optimized using the inter-frame spatial dependency. The main contributions of our work are:

1. The introduction of inter-frame flattening to reduce the curvature in the fovea and further improve the robustness of the algorithm;
2. The time complexity is greatly reduced by using inter-frame or intra-frame information which limits the search region;
3. A better distinction is attained for the boundaries closely located by applying biasing and masking techniques in the same search region;
4. A reduced number of nodes in the graph by down-sampling pixels in the lateral direction of the search region.

As a result, the processing speed for each frame has been greatly improved by about 10 times as compared to the previous work by Chiu et al. [17] and the processing of the whole OCT volume data (644 × 496 × 51 voxels) can be finished within 26 seconds. Up to the authors’ best knowledge, the speed of OCTRIMA 3D outperformed the existing works reported so far [17, 21, 22]. The segmented boundaries in each B-scan were combined to form smooth surfaces and were compared with three state-of-the-art graph search based segmentation algorithms [17, 21, 22]. In addition, segmentation results were compared to a ground-truth, which is the manual delineation of retinal boundaries. Two graders provided the manual labelings and inter-observer differences are used as benchmark to evaluate the accuracy. The results showed that OCTRIMA 3D is more close to the manual labeling as compared to the IOWA reference algorithm and Dufour’s Algorithm. The accuracy of OCTRIMA 3D is similar to that reported by Chiu et al. [17] but our implementation is much faster. Experiments to compare with manual labeling were conducted on 100 OCT B-scans from 10 healthy subjects and the average unsigned error obtained for eight surfaces was about 1 pixel. The absolute detection error on each surface is found to be significantly smaller than the inter-observer difference (*p* < 0.001).

## Methods

This section is organized as follows: first, the boundary detection framework is described in Section 1; more implementation details for OCTRIMA 3D are presented in Section 2; Volumetric scans from Spectralis SD-OCT (Heidelberg Engineering GmbH, Heidelberg, Germany) are used to evaluate the performance of OCTRIMA 3D as described in Section 3; using the manual labeling as the ground truth, the algorithm is also compared with the IOWA Reference Algorithm [21], Dufour’s algorithm [22] and Chiu et al. [17] as discussed in Section 4; the performance metrics are presented in Section 5.

### 1. Shortest-Path Based Graph Search for Boundary Detection

**Fig 1:**
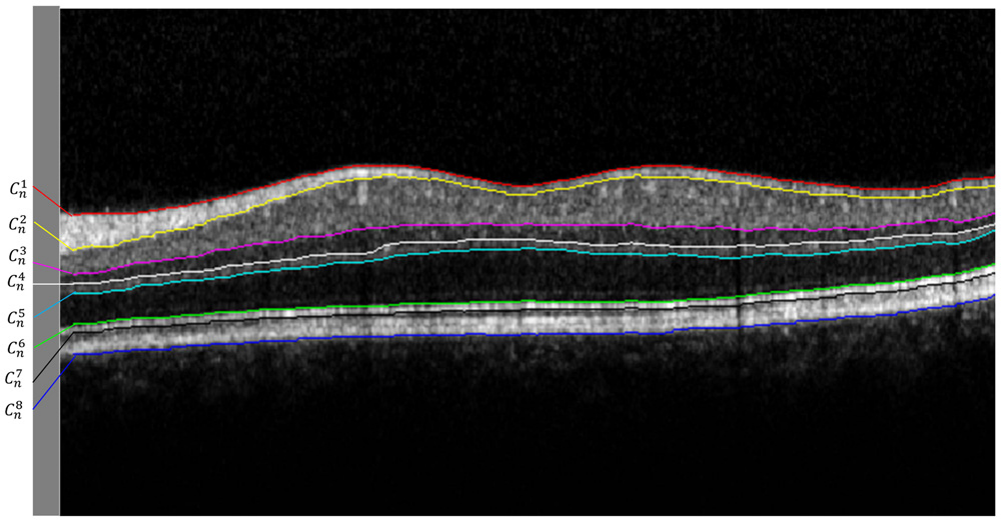
Exemplary OCT B-scan from Spectralis SD-OCT showing eight intraretinal layer boundaries labeled as $C_{n}^{1}$, $C_{n}^{2}$… $C_{n}^{8}$. Note that boundaries are delineated with red, yellow, magenta, white, cyan, green black and blue solid lines, respectively and the notations are summarized in Table 1. A parafoveal scan is chosen in order to show all the layers that are segmented by OCTRIMA 3D.

In this study, a total of eight retinal layer boundaries that were clearly visible were targeted for analysis. These boundaries are illustrated in Fig 1 and their corresponding notations are summarized in Table 1.

**Table 1:** Notations for eight target boundaries, n denotes the frame number.

Notations	Name of the boundary
$C_{n}^{1}$	internal limiting membrane (ILM)
$C_{n}^{2}$	outer boundary of the retinal fiber layer (RNFL_*o*_)
$C_{n}^{3}$	inner plexiform layer-inner nuclear layer (IPL-INL)
$C_{n}^{4}$	Inner nuclear layer-outer plexiform layer (INL-OPL)
$C_{n}^{5}$	outer boundary of the outer plexiform layer (OPL_*o*_)
$C_{n}^{6}$	inner segment-outer segment (IS-OS)
$C_{n}^{7}$	outer segment-retinal pigment epithelium (OS-RPE)
$C_{n}^{8}$	retinal pigment epithelium-choroid (RPE-CH)

The framework proposed by Chiu et al. [17] to segment retinal boundaries in each frame was used in this study. For completeness, the model is briefly presented in this section.

The problem of boundary detection in a given normalized gradient image *g* is modeled as finding the minimum weight path or the shortest-path in graph *G* = (*V*,*E*), where *V* is a set of vertices and *E* is a set of weighted undirected arcs. The weight of edges are positive numbers and zero-weight indicate non-connected edges. To make the end point initialization fully automatic, two additional columns are added on both ends of the gradient image and the gradient value of the two virtual columns are set to 1. Each pixel in the conjunct gradient image *g* ^*c*^ is represented by a vertex and each vertex in the graph is only connected with the eight nearest pixels on the sides and corners. The weights of the arcs are calculated based on the gradient value as

$$w\left(a,b\right)=\{\begin{array}{cc} 2-\left(g_{a}^{c}+g_{b}^{c}\right)+w_{min} & \;\text{if}|a-b|\leq \sqrt{2}\\ 0 & \;\text{otherwise} \end{array}$$ (1)

where **a** and **b** denotes two distinct elements of *V* and *w*_*min*_ is a small stabilization constant [17]. The pixels with higher values in the gradient image have smaller weights on the connecting arcs and hence have better chances of being selected. The most prominent boundary is detected as the minimum weight path from the first to the last vertex in *V* using Dijkstra’s Algorithm [23].

The framework using the shortest-path based graph search is able to detect only one boundary for each graph. For multiple boundaries, careful search region refinement is needed. For example, the connectivity-based segmentation is employed in [17] for search region refinement when segmenting intraretinal layers. In terms of graph constriction, it means that the connecting arcs outside of the search region have to be removed before the shortest-path search. The algorithm in [17] was tested on 100 OCT B-scans obtained from 10 healthy subjects. The average thickness error reported for various retinal layers was about 1 pixel and the average processing time for each frame was 9.74 seconds [17].

**Fig 2:**
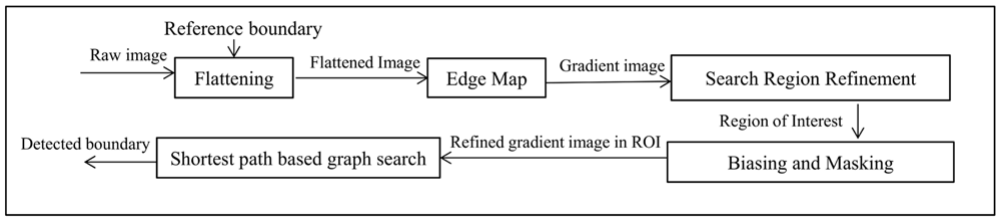
OCTRIMA 3D framework to detect each intraretinal layer boundary using the shortest-path based graph search approach.

In this work, OCTRIMA 3D, a real-time automatic algorithm to segment eight retinal boundaries in OCT volume data is developed based on the aforementioned framework. We proposed a new framework to detect each boundary using the shortest-path graph search as shown in Fig 2. Particularly, in order to detect each boundary, flattening is first performed to reduce the curvature of the target boundary. Second, a reference boundary is used in the alignment process to facilitate the flattening procedure. Then, the flattened image is convolved with edge kernels to calculate the gradient image. Of note, the selection of the edge kernel depends on the orientations of the target boundary. Next, the search region is refined using the location of the previous detected boundary in the current frame or the previous frame. In this paper, the term search region or region of interest (ROI) refers to the rectangle area in the OCT image that could possibly contain the target boundary. Biasing or masking are needed when more than one boundary are located in the same ROI. In the last step, a new graph is constructed using the down-sampled gradient image in the ROI and only the pixels in the search region are included in the vertices set. Finally, the result of the shortest-path search method is interpolated to obtain the target boundary.

#### 1. 1 Flattening

**Fig 3:**
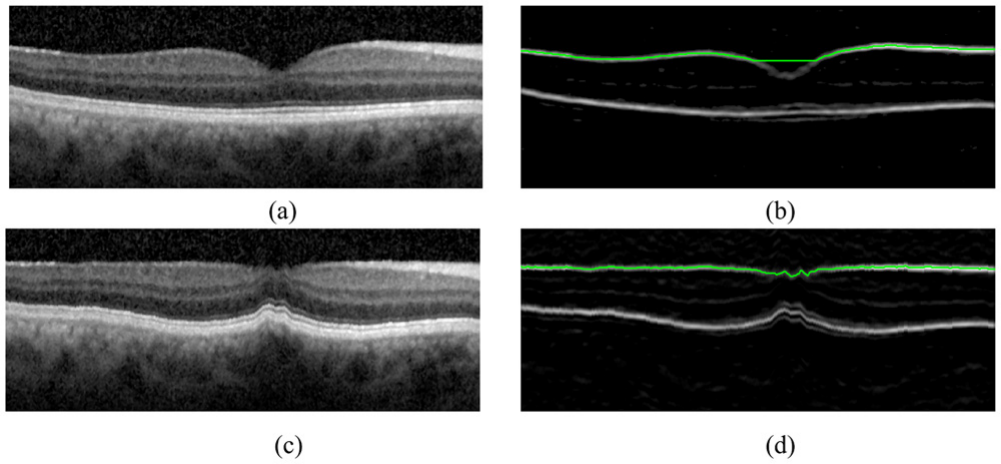
The shortest-path based graph search methodology prefers a geometric straight line and may fail to delineate the ILM boundary in the central region of the fovea. The ILM boundary can be detected correctly when the flattening operation uses the ILM border from a previous frame. Note that (a) and (c) are the raw OCT scans at the fovea and the resulting flattened image, respectively. (b) and (d) shows the results of the ILM boundary detection in (a) and (c).

Flattening is defined as the step that shifts A-scans up and down to make the reference boundary flat and is a commonly used preprocessing step in OCT segmentation tasks [16, 17]. As fewer nodes leads to a lower total weight, the graph search algorithm prefers geometric short path. Hence, horizontal boundaries with less curvature are better delineated in the image. The reference boundary is normally parallel to the target boundary and is detected in the earlier steps. The IS-OS boundary in the current frame is usually chosen as the reference boundary to remove the effect of retinal curvature. However, the ILM in the fovea region still has big curvature after flattening with the IS-OS border and may cause the boundary detection algorithm fail as shown in Fig 3(a) and 3(b).

In this work, we have improved the flattening of the ILM boundary by exploiting the smoothness of the retinal surface between adjacent B-scans. More specifically, the reference boundary for the ILM is chosen as the corresponding boundary in the previous frame to improve the robustness of the algorithm. After flattening, the ILM boundary has reduced curvature in the central region of the fovea and it can be correctly detected as shown in Fig 3(c) and 3(d). In this paper, the flattening process is named as *inter-frame flattening* if the reference boundary is on the previous frame. Otherwise, the flattening process is called *intra-frame flattening*.

#### 1.2 Edge Map

The gradient value of each pixel indicates the possibility of belonging to a boundary and the calculation of the gradient image is essential in the graph search algorithm. Because the speckle induced high gradient values are randomly located and not connected, the graph search algorithm could easily distinguish them from the actual boundary. Advanced denoising techniques, such as anisotropic diffusion or nonlinear complex diffusion [9, 10, 12, 13], are not needed. As the boundaries in retinal layers are usually horizontal after the flattening step, we only consider two orientations, dark-to-bright and bright-to-dark.

To detect the boundaries with dark-to-bright transition, the gradient image is calulated as

$$g=k_{d2b}*I,$$ (2)

where *I* is the B-scan image and the convolution kernel is defined as

$$k_{d2b}=\left[l_{d2b}\;l_{d2b}\;l_{d2b}\;l_{d2b}\;l_{d2b}\right],$$ (3)

and

$$l_{d2b}={\left[1\;1\;1\;1\;1\;0\;-1\;-1\;-1\;-1\;-1\right]}^{T}.$$ (4)

After convolution, the gradient values which are less than 0 are set to zeros and all the gradient values are normalized to the range between 0 and 1. We assumed that the gradient value near the retinal boundary followed the step edge model [24] and the convolution kernel was designed as a matched filter. The size of the kernel is determined experimentally so that the speckle noise is reduced by averaging with the neighboring pixels without losing much details in the horizontal direction.

For the boundaries with bright-to-dark transition, kernel *k*_*b*2*d*_ = −*k*_*d*2*b*_ is used.

#### 1.3 Search Region Refinement

In order to detect multiple boundaries with the same orientation, the search region could be limited to a different region of interest (ROI). Both the intra-frame and the inter-frame search region refinement were used to locate the ROI.

For the intra-frame search region refinement, the search region is defined according to the relative position of the boundaries within the same image. For example, taking into account that the IPL-INL boundary is always located between the ILM and IS-OS boundaries, the search region of $C_{n}^{3}$ was selected as the rectangle area between $C_{n}^{1}$ and $C_{n}^{6}$.

**Fig 4:**
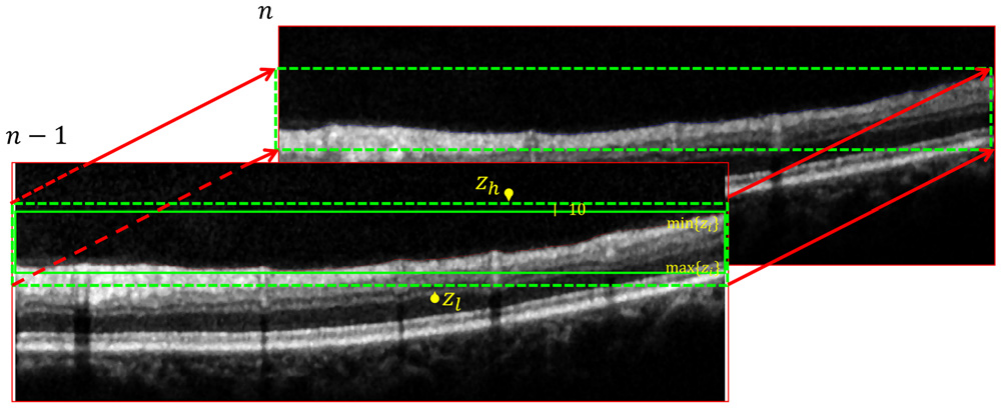
Illustration of the search region refinement using the inter-frame dependency approach. Taking into account that the ILM boundary is delineated in the frame *n* − 1, the search region of $C_{n}^{1}$ could be limited to be $\text{min}\{(z^{i}\}-10,\text{max}\{z^{i}\}+10],\forall (x,z^{i})\in C_{n-1}^{1}$.

For the inter-frame search region refinement, the boundary location of the frame *n* − 1 was used to refine the search region in the frame *n* as illustrated in Fig 4. Our assumption is that the difference of the ILM’s axial position between adjacent frames are less than 10 pixels. Hence, we could limit the search region of $C_{n}^{1}$ to [*Z*_*h*_,*Z*_*l*_], where

$$Z_{h}=\text{min}\left\{z^{i}\right\}-10,Z_{l}=\text{max}\left\{z^{i}\right\}+10,\forall \left(x,z^{i}\right)\in C_{n-1}^{1}.$$ (5)

In our implementation, only the pixels in the ROI were used as the vertices of the graph *G*. However, the original framework proposed by Chiu et al. [17] used every pixel of the B-scan to construct the graph and only removed the vertices out of the ROI for each boundary detection task.

As it will be discussed in Section 1.5, the time complexity of the algorithm is a function of the number of vertices. Hence, the processing speed is improved when compared with [17] as the number of vertices is greatly reduced in OCTRIMA 3D.

#### 1.4 Biasing and Masking

**Fig 5:**
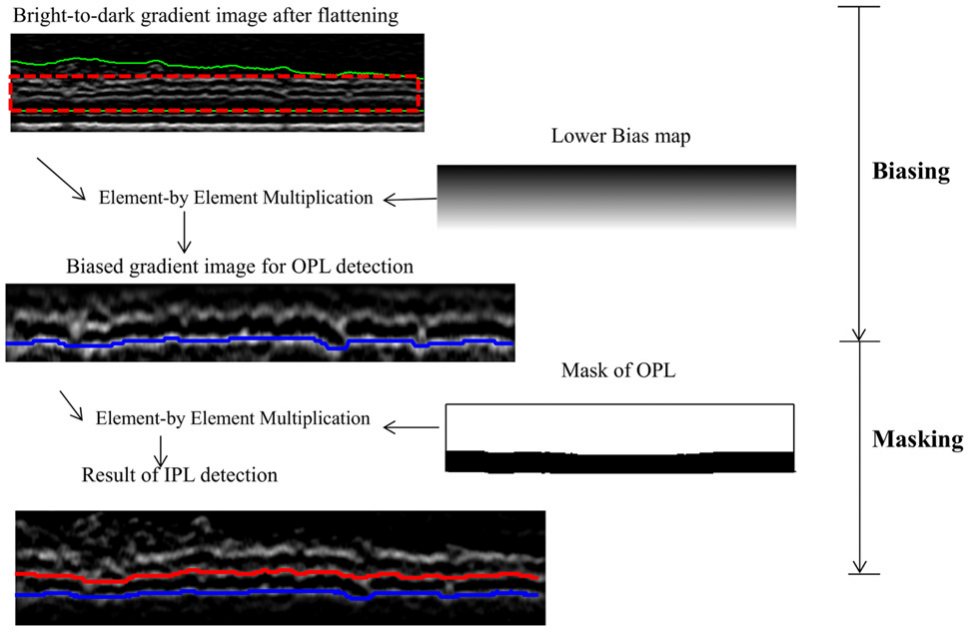
Illustration of the biasing and masking operations for the boundary detection of the IPL-INL (red) and OPL_o_ (blue). The search region or ROI (red dotted rectangle) is the area between IS-OS and ILM (green solid lines). After element-by-element multiplication with lower bias map, the OPL_o_ is more prominent in the gradient image and can be detected easily. A binary mask is generated to set all the pixels below OPL_o_ to zeros and the second lowest boundary, IPL-INL, is detected using the shortest-path graph search.

Biasing and masking are introduced to detect multiple boundaries located in the same ROI. For example, the IPL-INL border and OPL_o_ are closely located and both are characterized by a dark-to-bright transition with low contrast, therefore it is difficult to distinguish them with the graph search automatic algorithm. In this circumstance, biasing and masking are applied to delineate these two boundaries using the relative position of the boundaries as illustrated in Fig 5. This technique is explained as follows:

- **Biasing**
  It is assumed that OPL_o_ is always below the IPL-INL border and that both boundaries are between the IS-OS and ILM boundaries (green solid line in Fig 5). The ROI is the area between the IS-OS and the lowest point of the ILM as indicated by the red dashed rectangle in Fig 5 The flattened bright-to-dark gradient image in the search region is first multiplied with a bias map *B* ^*l*^ defined as
  $$B_{z,x}^{l}=\frac{z-1}{M-1},$$ (6)
  where *M* is the number of rows in the region of interest and (z, x) denotes the position in the axial and lateral directions. After biasing, the lowest boundary, i.e. the OPL_o_, has better contrast than the other layers. By using the shortest-path based graph search, the OPL_o_ could be detected automatically.
- **Masking**
  Masking refers to the element-by-element multiplication between the gradient image in the search region and a mask image when detecting the second lowest boundary, i.e. the IPL. To create the mask image, the pixels that are lower than the previously detected boundary are set to zeros and the other pixels are set to ones. After the element-by-element multiplication step, the gradient values in the pixels below the lowest boundary are zeros and hence the algorithm could detect the second lowest boundary using shortest-path based graph search.

As the gradient values in the search region are reduced after the biasing and masking operation, the intensities of the gradient image in each column of the ROI are normalized to [0, 1] to improve the contrast when biasing and masking is needed.

#### 1.5 Shortest-Path Based Graph Search

Once the flattening, search region refinement, and biasing and masking procedures are performed, the pixels’ intensity values in the ROI *g*_*M* × *N*_ indicate the likelihood for detecting a potential boundary. The detection of the specific boundary is formulated as finding the shortest path as described earlier. The constructed graph is highly sparse and every vertex has eight connecting arcs only. Using Dijkstra’s Algorithm [23], the time complexity of the graph search method is *O*(*log*(∣*V*∣)*∣*E*∣), where ∣*V*∣ and ∣*E*∣ are the number of nodes and arcs [23]. In the context of our boundary detection framework, ∣*V*∣ = *MN* and ∣*E*∣ = 8*MN*. Hence the time complexity is *O*(*log*(*MN*)*MN).

In order to improve the processing time further, we have down-sampled the gradient image by a factor of 2 in the lateral direction. Because the retinal layers are smooth between adjacent columns, the reduction in the lateral resolution results in a great improvement in the processing speed without affecting accuracy much. The boundary location in the raw image is linearly interpolated from the detection results in the down-sampled image and further smoothed with a moving average filter.

### 2. OCTRIMA 3D

**Fig 6:**
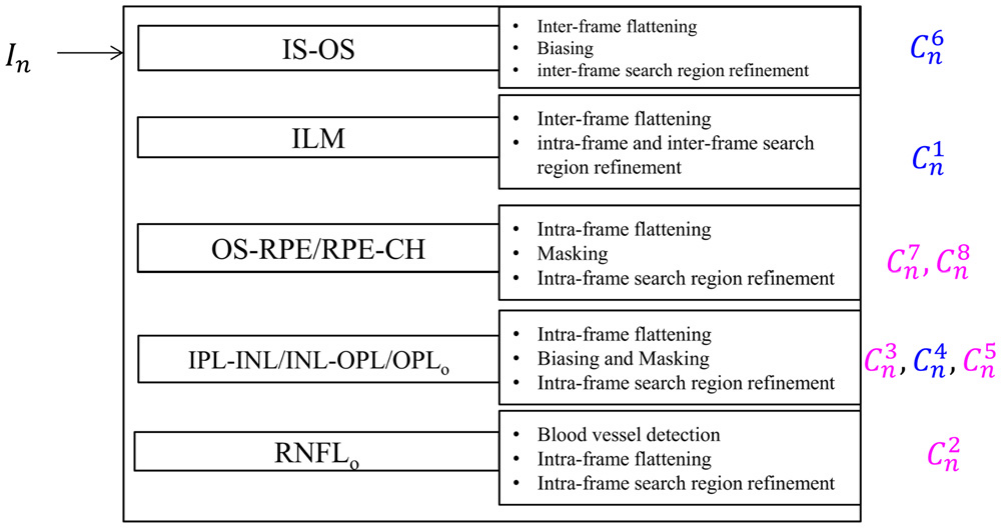
The overview of OCTRIMA 3D framework. The boundaries labeled using blue and red fonts have the dark-to-bright and bright-to-dark transitions, respectively.

This section describes the implementation details of OCTRIMA 3D for the detection of eight retinal boundaries. The overview of the method is shown in Fig 6.

#### 2.1 Detection of the IS-OS boundary

The IS-OS border is the most prominent and flat boundary in the retinal OCT B-scans of healthy subjects and it is detected on the dark-to-bright gradient images. The boundary detection strategies for the first frame and subsequent frames are different. For the first frame, the search region is the whole gradient image and a bias map *B* ^*l*^ is multiplied with the gradient image to eliminate the interference from the ILM which is a high contrast boundary. The result of the shortest-path based graph search is $C_{1}^{6}$. For the subsequent frames, the detection result of the ILM boundary in the previous frame is used for inter-frame flattening and inter-frame search region refinement.

#### 2.2 Detection of the ILM boundary

The ILM ($C_{n}^{1}$) border is another high contrast boundary on the dark-to-bright gradient image. Its detection method is described as follows:

For the first frame, the detected IS-OS ($C_{1}^{6}$) boundary is used as the reference boundary for flattening. Intra-frame search region refinement defines the area above $C_{1}^{6}$ as the search region for the ILM border. The result of the shortest-path based graph search is $C_{1}^{1}$. In order to detect the ILM boundary in the subsequent frames ($C_{n}^{1}$, *n* > 2), the inter-frame flattening (as illustrated in Fig 3) is applied to reduce the curvature of the ILM border in the central region of the fovea. The inter-frame search region refinement is used to reduce the processing time.

#### 2.3 Detection of the OS-RPE and RPE-CH boundaries

Intra-frame flattening is performed for the OS-RPE and RPE-CH boundaries detection using $C_{n}^{6}$ as the reference boundary. The edge kernels for the OS-RPE and RPE-CH boundaries are *k*_*b*2*d*_ and *k*_*d*2*b*_, respectively. The search region is the rectangle area with a height of 40 pixels below the flattened IS-OS edge in the current frame. The RPE-CH ($C_{n}^{8}$) boundary is detected using the shortest-path based graph search on the bright-to-dark gradient image. The masking operation is applied to set all the gradient values on the pixels below $C_{n}^{8}$ to zeros. The only boundary in the search region of bright-to-dark images is the OS-RPE ($C_{n}^{7}$) border which can be detected easily.

#### 2.4 Detection of the IPL-INL, INL-OPL and OPL_o_ boundaries

The intra-frame flattening is performed using $C_{n}^{6}$ as the reference boundary for the detection of the IPL-INL / INL-OPL / OPL_o_. The bright-to-dark edge kernel is used to detect the IPL-INL ($C_{n}^{3}$) and OPL_o_ ($C_{n}^{5}$) boundaries. Intra-frame search region is defined as the area between the flattened IS-OS border and the lowest point of the ILM boundary in the current frame. As the separation between the IPL-INL and OPL_o_ is small and they are in the same search region, masking and biasing operations are performed as described in Section 1.4. As for the INL-OPL ($C_{n}^{4}$) border’s detection, the dark-to-bright edge kernel is used to filter the flattened image. The search region is the same as the one used in the detection of the IPL-INL and OPL_o_. To make sure the INL-OPL border is always between the IPL-INL and OPL_o_, the gradient value on the pixels that are above the IPL-INL and below the OPL_o_ are set to zeros by using the masking operation. The shortest-path graph search could delineate the INL-OPL boundary easily.

#### 2.5 Detection of the RNFL_o_ boundary

**Fig 7:**
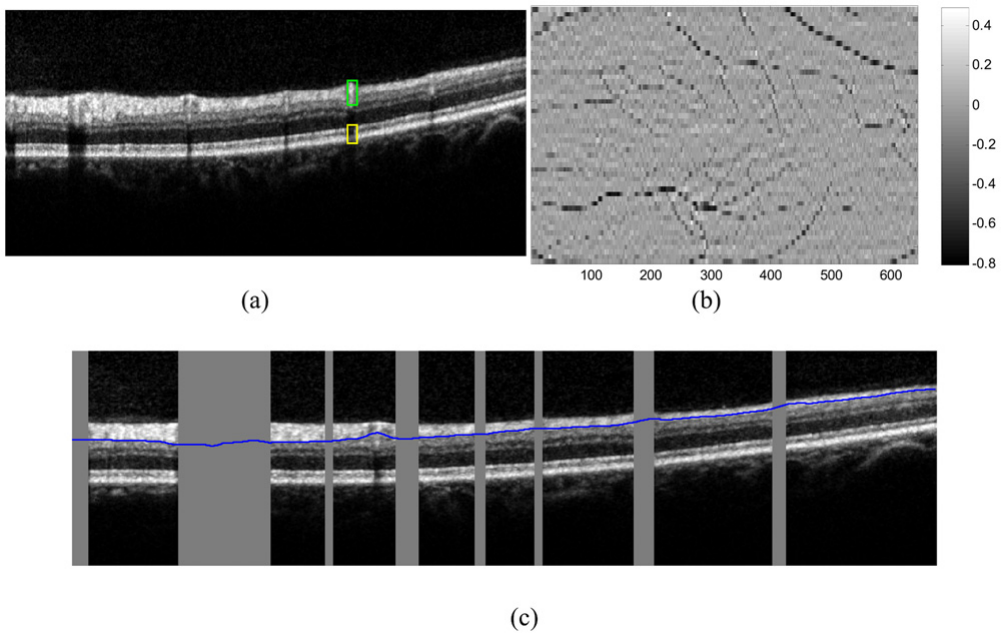
Detection of A-scans affected by retinal blood vessels. (a)The shadowing effect from the retinal blood vessels is more pronounced near the RPE (yellow rectangle, note the hypo reflective regions) and less pronounced near the ILM (green rectangle). (b) An example of the enface map of a macular volume OCT. (c) The result of RNFL_o_ detection (blue solid line). The regions where the A-scans are affected by the blood vessel shadowing is highlighted with gray rectangles.

The disruption of the RNFL’s outer boundary (RNFL_o_) caused by the presence of the blood vessels on the retina poses a challenge for the graph search algorithm. In our study, we detect the A-scans affected by the blood vessels by segmenting the enface map of the OCT volume data. As shown in Fig 7(a), the blood vessels have altered the distribution of A-scans in two ways: (1) the intensities of the pixels just below the ILM layer are higher than the surroundings A-scans and; (2) the intensity of the pixels between the IS-OS and RPE-CH boundaries is lower than in the neighboring pixels. The enface map is defined as:

$$E_{n,x}=\frac{1}{z_{8}-z_{6}+1}\sum_{z=z_{6}}^{z_{8}}I_{z,x}^{n}-\frac{1}{30}\sum_{z=z_{1}}^{z_{1}+29}I_{z,x}^{n},$$ (7)

where *I* ^*n*^ denotes the *n*th B-scan/frame in the volume, $(x,z_{1})\in C_{n}^{1}$,$(x,z_{6})\in C_{n}^{6}$ and $(x,z_{8})\in C_{n}^{8}$. The A-scan that is affected by the blood vessels would have higher value of *E*_*n*,*x*_ and the location of blood vessels is determined by thresholding the enface image with the empirically selected value of −0.1. An example enface map is given in Fig 7(b).

Once the location of the blood vessels is determined, the outer boundary of the RNFL ($C_{n}^{2}$) is detected as follows: First, the raw image is flattened with the ILM boundary as the reference boundary ($C_{n}^{1}$) and the *k*_*b*2*d*_ is used as the filtering kernel. Then the search range is defined between the flattened ILM edge and the highest point of the IPL border. To overcome the disruption caused by the blood vessels, the gradient values on the A-scans affected by the blood vessels are set to ones. Hence, the weight of the arcs in the blood vessel region is equal to *w*_*min*_, which is similar to the algorithm proposed in [17]. The result of the outer RNFL detection and vessel shadow location are illustrated in Fig 7(c).

After the eight intraretinal boundaries are segmented, the detected boundaries $C_{n}^{i}$ form 8 surfaces *B* ^*i*^,*i* = 1,2…8 defined as

$$B_{m,n}^{i}=z_{n}^{i}\left(m\right),\forall \left(m,z_{n}^{i}\left(m\right)\right)\in C_{n}^{i},$$ (8)

where $z_{n}^{i}(m)$ is the location of *i*th boundary on *m*th column and *n*th frames.

### 3. Clinical Data

We conducted our study on 10 Spectralis SD-OCT (Heidelberg Engineering GmbH, Heidelberg, Germany) volume data sets from 10 healthy adult subjects. The Institutional Review Board of the University of Miami approved the study. The research adhered to the tenets set forth in the declaration of Helsinki and written informed consent was obtained from each subject. The healthy subjects were selected based on a best-corrected visual acuity of at least 20/25, a history of no current ocular or systematic disease, and a normal appearance of the macula when examined with contact lens biomicroscopy.

Each subject was scanned using IR+OCT scanning mode with a 30^°^ area setting. The captured volumes contained 61 images with the dimensions of 768 × 496 pixels (width× height). The axial resolution was 3.9 microns and the transversal resolution varied from 10 to 12 microns. The inter B-scan spacing was from 120 microns to 140 microns. To reduce the speckle noise and enhance the image contrast, every B-scan was the average of five aligned images using the TruTrack active eye tracking technology [25] (*ART* = 5). We exported the volume scans from Spectralis SD-OCT device using the built-in xml export format, which consisted of 61 JPG images and an xml file specifying the volume scanning details.

In addition, experiments were also conducted on 100 SDOCT images obtained with the Bioptigen device (Bioptigen Inc, Morrisville, North Carolina, USA) images from 10 subjects. The data and the manual labelings were kindly provided by Chiu et al. and the details can be found in [17].

Besides the OCT data from healthy subjects, two B-scans from subjects with pathologies were also used to explore the potential of OCTRIMA 3D in pathological cases. One scan was obtained from a patient with diabetic macular edema (DME) captured at the Bascom Palmer Eye Institute, University of Miami. The other B-scan was from an eye with dry age-related macular degeneration (drye-AMD) downloaded from Dufour’s software package’s website [22].

### 4. Experimental Setup

OCTRIMA 3D was implemented using Matlab R2014a on a computer with the CPU of Intel Core i7-2600@ 3.4 GHz 3.4 GHz. Prior to the 8 boundaries segmentation procedure, the ILM and RPE-CH borders were segmented from frames 21 to 40 and the point with the smallest distance was detected as the fovea. No training was needed in this work. The OCT A-scans outside the 6mm × 6mm (lateral × azimuth) area and centered at the fovea were cropped to remove low signal regions.

In order to evaluate the performance of OCTRIMA 3D, we compared our segmentation results with three existing graph-based segmentation approaches and the results from a manual grader using the following three experiments:

- Comparison between Dufour’s algorithm, IOWA Reference Algorithm and OCTRIMA 3D: Dufour’s software is able to read the xml built-in format and detect 6 surfaces from the volumetric data automatically. The segmented surfaces were saved into a.csv file.
  In order to be able to use the Iowa reference algorithm, all scanned data in the xml format was converted to.vol raw format by our customized program using the following steps: 1. read the template.vol file from [26] using the Matlab script [27]. 2. Convert the intensity values in the xml format to the raw format by taking the fourth power [28]. 3. Replace the image data in the template.vol file with the converted raw data obtained in the second step. The converted.vol file was loaded into the IOWA Reference Algorithm and segmented by the “10 Layer Segmentation of Macular OCT” function. Eleven surfaces were segmented fully automatically and saved into a surface.xml file.
  The detected six surfaces from Dufour’s software are corresponding to ILM, RNFL_o_, IPL-INL, OPL_o_, IS-OS and RPE-CH in OCTRIMA 3D and are equivalent to surface 1, 2, 4, 6, 7 and 11 in the IOWA Reference algorithm, respectively.
  We compared the 6 segmented surfaces from the IOWA Reference Algorithm, Dufour’s software and OCTRIMA 3D in the ETDRS regions using the manual labeling from an expert grader as the ground truth qualitatively and quantitatively. Comparison was made by paired t-test and the level of significance was set at 0.001.
- Comparison with the algorithm developed by Chiu et al. [17]: To validate OCTRIMA 3D, we segmented the same set of SDOCT images obtained with the Bioptigen device (Bioptigen Inc, Morrisville, North Carolina, USA) with the algorithm by Chiu et al. The manual labelings from their study reported in [17] were used as the ground truth for comparison. This comparison was performed to mainly assess the potential operational time’s improvement.
- Comparison with manual graders’ results: In order to estimate the accuracy of OCTRIMA 3D, 100 OCT B-scans from 10 healthy subjects were used in the manual labeling experiment. A subset of 10 images were randomly selected from every volumetric data of a patient for labeling and at least two of these frames contained the fovea. Tracking boundaries of the retinal layers manually is a time-consuming process. In this study, we designed a software tool using Matlab 2014a for manual labeling. Particularly, once the observer or grader clicked on the points along each border, the manual tracing resulting from linear interpolation between the clicked points was taken as the final ground truth for comparison. The grader could also move, add and delete the clicked points to modify the boundary tracings. The labeling task was performed with extreme carefulness by two observers, Observer 1 and Observer 2. On average, it took about 30 minutes to label one frame. The delineated results from Observer 1 were taken as the ground truth and the inter-observer difference were used as a benchmark to evaluate the accuracy. Comparison was made by paired t-test and the level of significance was set at 0.001.
- Potential application on retinal images showing pathological features: Detection of pathological retinal structures is a difficulty of countless everyday clinical importance. Two OCT B-scans from patients with retinal pathologies as described in the Section 3 were used to explore the potential of extending OCTRIMA 3D to segment volume data showing pathological feautures.

### 5. Performance Metrics

The following performance metrics were defined to objectively measure the difference between the detection results ($B_{m,n}^{i}$) and ground truth (denoted as ${\bar{B}}_{m,n}^{i}$):

- The signed error (SE) between the automatic detection and ground truth are defined by
  $$SE=\left(MSE\pm SSE\right),MSE=\mu \left(B_{m,n}^{i}-{\bar{B}}_{m,n}^{i}\right),SSE=\sigma \left(B_{m,n}^{i}-{\bar{B}}_{m,n}^{i}\right),$$ (9)
  where *μ* and *σ* denote the mean and standard deviation of the matrices, respectively. The value of mean signed error (MSE) and standard deviation of signed error (SSE) indicate the bias and variablity of the detection results.
- The mean of the unsigned errors (MUE) which measures the absolute difference between the automatic detection results and manual labeling is defined by
  $$MUE=\mu (|B_{m,n}^{i}-{\bar{B}}_{m,n}^{i}|).$$ (10)
- The 95th percentile unsigned errors, denoted as *E*_95_, is the highest value of the unsigned error after removing the top 5% of the biggest values. It measures the upper bound of unsigned error except the extreme cases.

The run time for the eight boundaries segmentation procedure of the whole volume is measured to calculate the time complexity of the algorithm.

## Results

**Fig 8:**
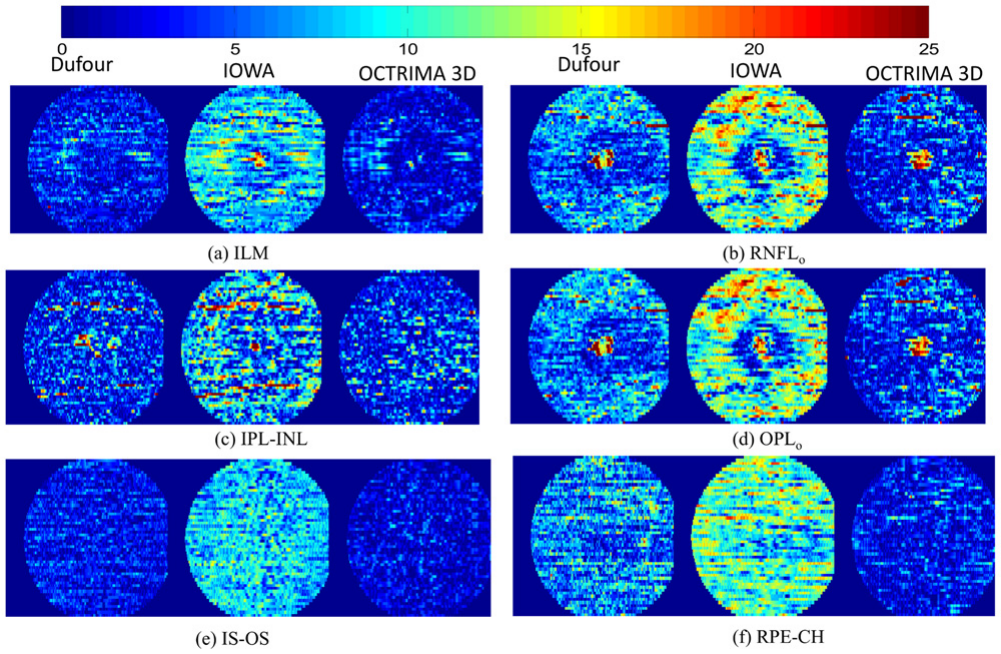
Comparison of unsigned segmentation errors on six surfaces between Dufour’s algorithm (left column), the IOWA reference algorithm (middle column) and OCTRIMA 3D (right column) in the ETDRS regions. The graph bar scale indicates the error magnitude in microns. The mean unsigned segmentation errors are reported in Table 2.

**Fig 9:**
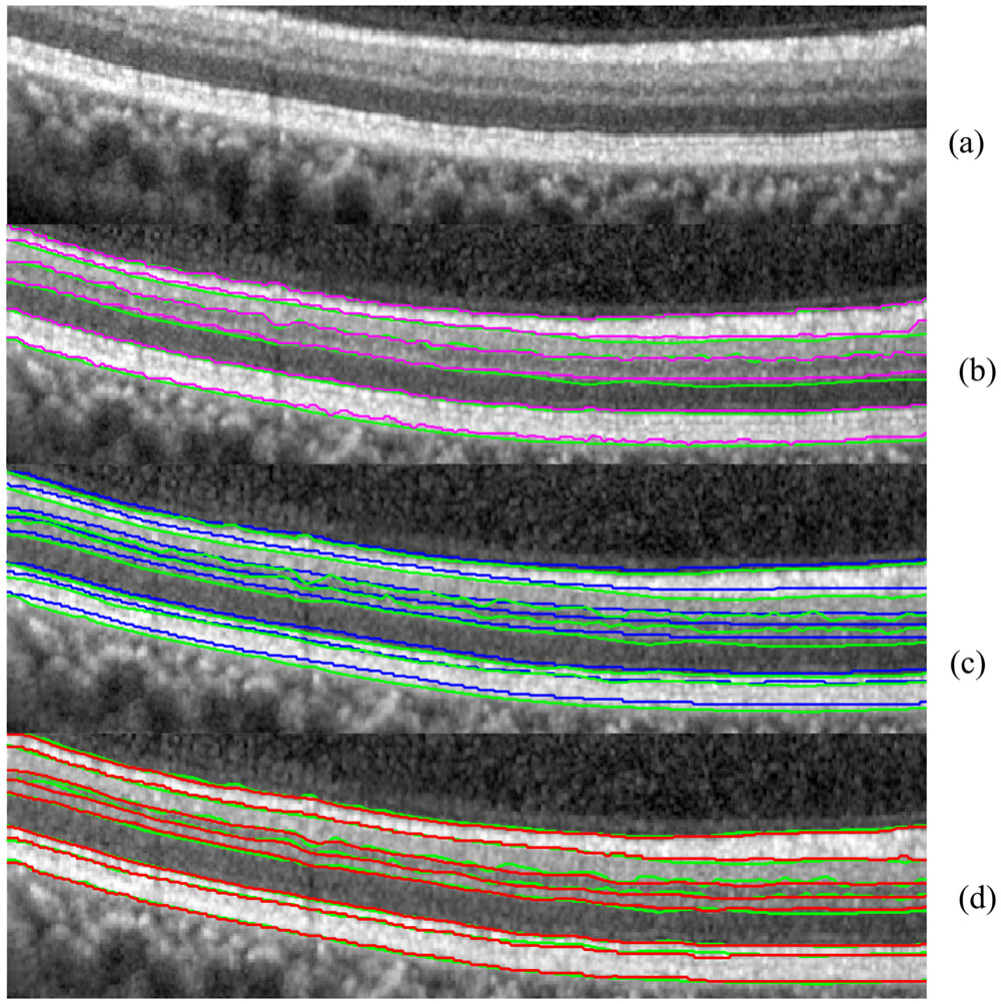
The comparison between Dufour’s Software (magenta solid line), IOWA reference algorithm (blue solid line) and OCTRIMA 3D (red solid line) using manual labeling as the ground truth (green solid line).

**Fig 10:**
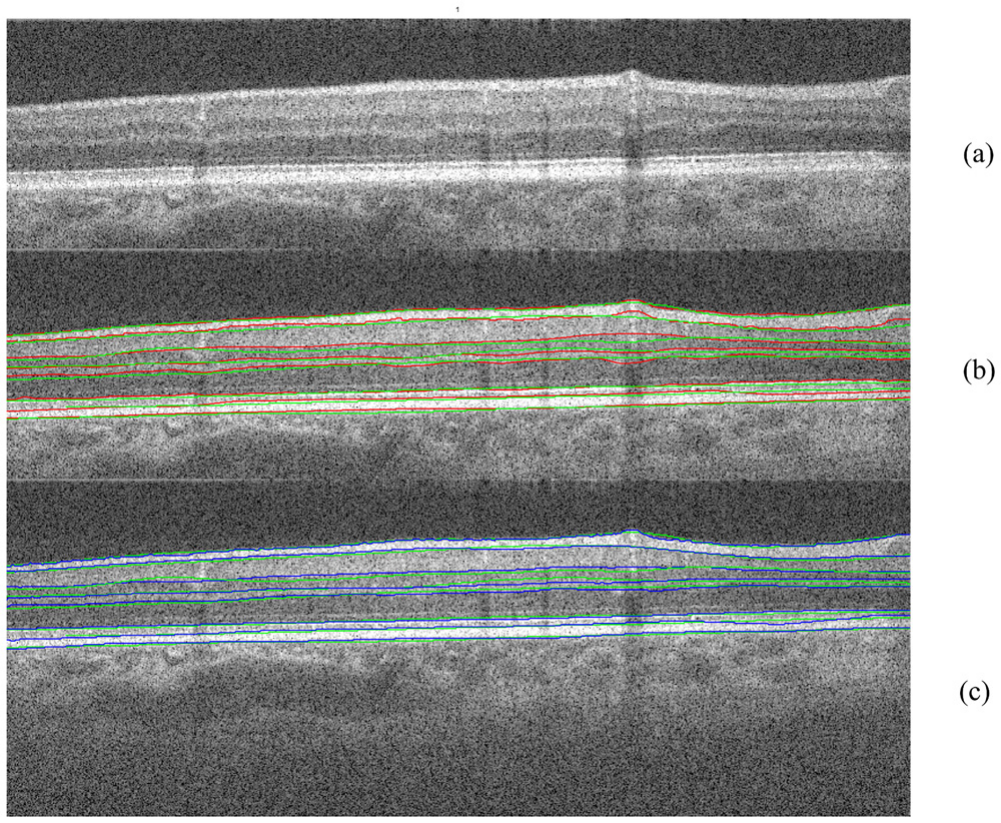
The comparison between OCTRIMA 3D (red solid line) and the algorithm by Chiu et al. (blue solid line) using manual labeling as the ground truth (green solid line).

**Fig 11:**
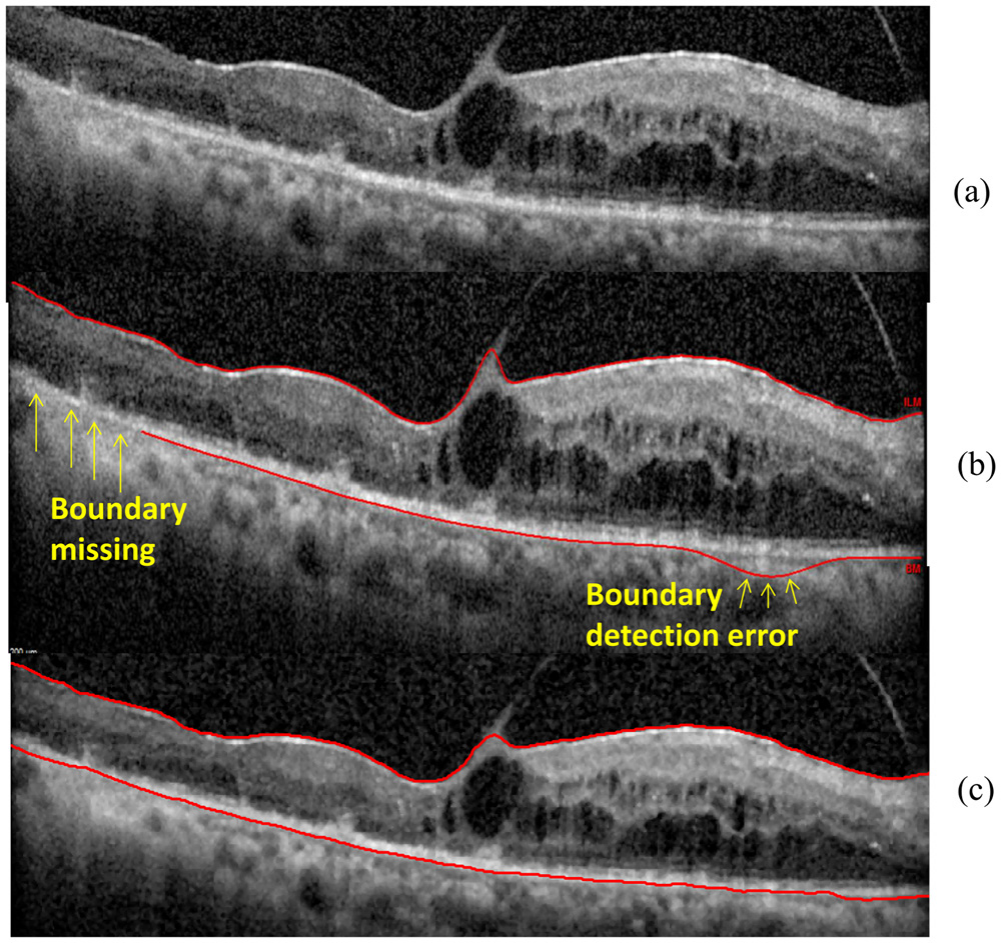
Algorithms performance in the B-scan obtained from the patient with diabetic macular edema. (a) The raw OCT B-scan. (b) The boundaries delineated by the built-in Spectralis SD-OCT software for the ILM and RPE-CH. The yellow arrows are indicating the boundary detection errors by the built-in software of the Spectralis device. (c) The boundaries delineated by OCTRIMA 3D for the ILM and the RPE-CH.

**Fig 12:**
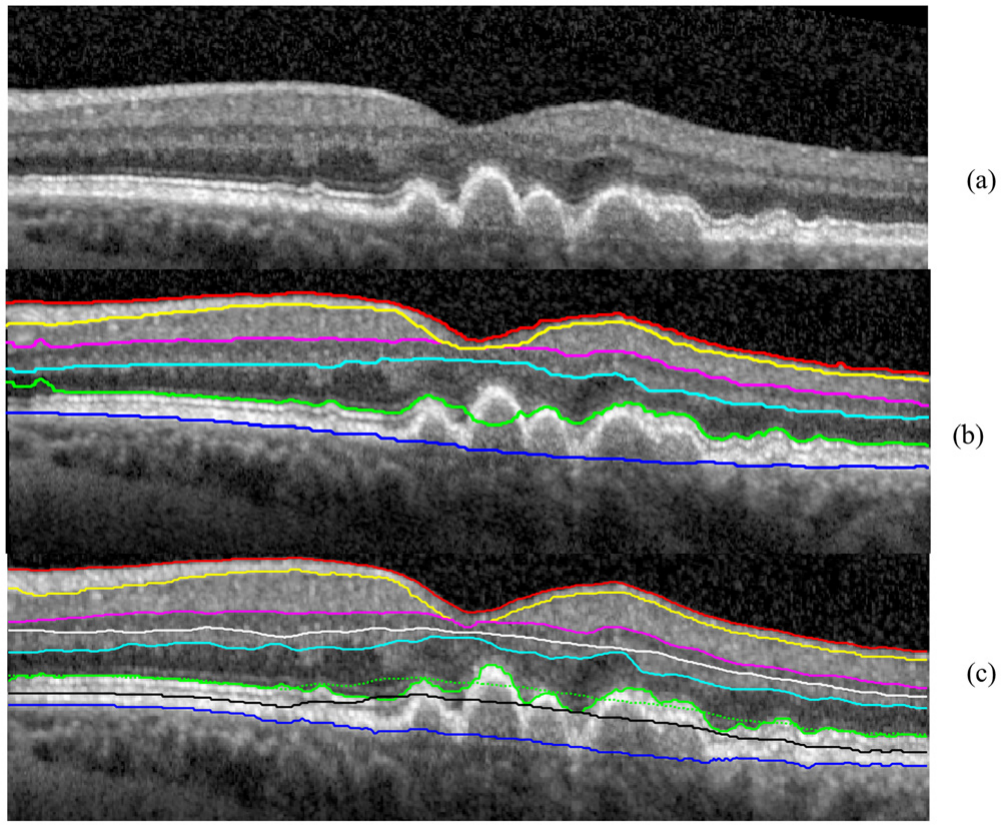
The segmentation results obtained for the B-scan in the eye with dry age-related macular degeneration using Dufour’s software and the OCTRIMA 3D algorithm. The legend of the boundaries is the same as Fig 1. (a) THe raw OCT B-scan. (b) The segmentation result of Dufour’s software. The IS-OS delineation failed at the left most and center area of the B-scan. (c) The initial segmentation results of OCTRIMA 3D detected retinal boundaries reliably except for the IS-OS in the drusen area (green doted line). By adjusting the flattening step, the IS-OS is delineated correctly (green solid line).

- **Results of the comparison between Dufour’s Software, the IOWA Reference Algorithm and OCTRIMA 3D**
  The output csv file from Dufour’s software was imported using Matlab. To interpret the results of the 10 layer segmentation procedure from the IOWA reference algorithm, the surface.xml file was read with a customized Matlab script. The segmentation procedures using OCTRIMA are illustrated in S1 Video. The processing time for the volume on the 6*mm* × 6*mm*(*lateral* × *azimuth*) area using our algorithm was 26.15 seconds while the processing time for Dufour’s software and the Iowa Reference Algorithm was about 60 seconds and 75 seconds, respectively. The unsigned detection errors obtained for six retinal surfaces are shown in Fig 8(a)–8(f). The average unsigned errors are shown in Table 2. As it can be seen, the unsigned error for OCTRIMA 3D in all the surfaces is significantly smaller than Dufour’s Software and IOWA Reference Algorithm (*p* < 0.001). The ILM, IS-OS and RPE-CH surfaces were more reliably delineated than the other three surfaces. As an example, the delineated boundaries on an OCT B-Scan are shown in Fig 9.
- **Results of comparison between the algorithm by Chiu et al. [17] and OCTRIMA 3D**
  The results of our algorithm on OCT images obtained from the Bioptigen device (Bioptigen Inc, Morrisville, North Carolina, USA) were compared to the Chiu et al. algorithm and the results are shown in Table 3. Every OCT B-scan was processed independently and the average processing time was 1.15 seconds. The processing time for Chiu et al’s algorithm was reported as 9.74 seconds using a computer with a CPU of Intel Core 2 Duo at 2.53 GHz. Our implementation showed a significant improvement in the processing time. As expected (see Fig 10), the results from OCTRIMA 3D agreed well with the Chiu et al. algorithm and the main discrepancy was on the vessel shadow regions. The slight difference in accuracy between OCTRIMA 3D and Chiu et al’s algorithm is subjective to different observers. In particular, the manual labelings provide by Chiu et al. is very smooth. In OCTRIMA 3D, the delineated boundaries trace the small bumps and hence a slightly increase of error is observed.
- **Results of comparison between manual labelings from two observers and OCTRIMA 3D**
  The difference between OCTRIMA 3D and manual labeling is shown in Table 4.
  Note that without segmentation bias correction, the bias of the results from OCTRIMA 3D were less than 1 pixel for all the boundaries. The average absolute error, which was measured with MUE, was in the range of [0.72, 1.7] pixel. The boundary 1, 6 and 8 had better performance than the other 5 remaining boundaries. OCTRIMA 3D detection unsigned error was significantly lower than the inter-observer difference (*p* < 0.001) for all of the eight boundaries. The upper bound of detection errors was between 1.86 to 4.36 pixels.
- **Results of OCTRIMA 3D for segmenting retinal images showing pathological features** The segmentation results of OCTRIMA 3D are shown in Fig 11 and Fig 12. As it is shown in Fig 11, OCTRIMA 3D outperformed the built-in algorithm from Spectralis SD-OCT in detecting the ILM and RPE-CH boundaries. The built-in software of Spectralis has obvious detection errors and missing areas for the RPE-CH layer while OCTRIMA 3D was able to detect the ILM and RPE-CH boundaries accurately without any adjustment to the current algorithm. In the particular case of the AMD eye (see Fig 12(a)), the retinal layers were disrupted and posed a challenge to the automatic segmentation softwares. The segmentation results of Dufour’s software was shown in Fig 12(b). The IS-OS boundary detection failed in the left most and center area of the B-scan. In comparison, the OCTRIMA 3D was able to segment the layer correctly except for the IS-OS and OS-RPE boundaries in the drusen area where the surfaces were not flat. Therefore, the delineation of the IS-OS boundary was corrected by removing the flattening step and enhancing the edge map. After refinement, the IS-OS boundary could be detected precisely as shown in Fig 12(c). This particular improvement is an indication that OCTRIMA 3D could be further optimized to quantify morphological or pathological features on images that are not quite flat. However, the performance of OCTRIMA 3D on segmenting the OCT images with various pathologies is going to be investigated more thoroughly in the future.

**Table 2:** Comparison of average absolute detection error in unit of pixels and microns between Dufour’s algorithm, IOWA Reference Algorithm and OCTRIMA 3D in ETDRS region.

Surface	Surface	Dufour’s Software		IOWA’s Ref		OCTRIMA 3D	
No.	Name	pixels	*μm*	pixels	*μm*	pixels	*μm*
$C_{n}^{1}$	ILM	0.82	3.36	1.10	4.27	0.71	2.77
$C_{n}^{2}$	RNFL_o_	1.69	6.53	1.78	6.95	1.22	4.74
$C_{n}^{3}$	IPL-INL	1.15	4.48	1.03	4.03	1.02	3.98
$C_{n}^{5}$	OPL_o_	1.83	7.14	1.59	6.20	1.04	4.06
$C_{n}^{6}$	IS-OS	0.76	2.96	1.07	4.19	0.54	2.11
$C_{n}^{8}$	RPE-CH	1.62	6.31	1.70	6.65	0.75	2.95

**Table 3:** Comparison results between OCTRIMA 3D and the algorithm by Chiu et al. on Bioptigen OCT images. The error is quantified with (MSE± SSE, MUE, *E*_95_) in unit of pixels.

Surface	MSE± SSE		MUE		*E*_95_	
No.	OCTRIMA 3D	Chiu et al.	OCTRIMA 3D	Chiu et al.	OCTRIMA 3D	Chiu et al.
$C_{n}^{1}$	−0.60±1.14	0.34±1.24	1.00	1.00	2.56	2.20
$C_{n}^{2}$	−0.74±1.69	0.38±1.79	1.42	1.38	2.98	2.50
$C_{n}^{3}$	−0.27±1.90	−0.28±1.71	1.37	1.33	3.78	3.30
$C_{n}^{4}$	−0.008±2.2	0.53±1.87	1.73	1.55	4.00	3.60
$C_{n}^{5}$	−1.36±2.61	−1.01±2.30	2.28	1.94	5.58	5.60
$C_{n}^{6}$	−0.82±1.10	0.97±1.10	1.07	0.91	2.94	2.50
$C_{n}^{7}$	−0.99±1.67	−0.42±1.45	1.57	1.13	4.00	3.10
$C_{n}^{8}$	−0.66±1.59	−0.67±1.61	1.24	1.31	3.30	3.80

**Table 4:** Comparison results between OCTRIMA 3D and manual labelings from two graders. The manual labeling from Observer 1 is taken as the ground truth and the inter-observer difference is reported as a benchmark to evaluate the accuracy. The difference is evaluated using (MSE± SSE, MUE, and *E*_95_) in unit of pixels.

	Inter-Observer		OCTRIMA3D-Observer 1			Paired t-test	
Surface No.	MSE± SSE	MUE	*E*_95_	MSE± SSE	MUE	*E*_95_	*p* < 0.001
$C_{n}^{1}$	−0.52±1.11	0.97	2.44	0.00±0.94	0.72	1.86	Yes
$C_{n}^{2}$	−0.63±1.76	1.31	3.55	-0.12±1.67	1.09	3.00	Yes
$C_{n}^{3}$	−0.89±1.54	1.42	3.45	0.46±1.19	0.97	2.51	Yes
$C_{n}^{4}$	0.13±1.64	1.30	3.21	0.36±1.32	1.00	2.72	Yes
$C_{n}^{5}$	−0.35±1.76	1.39	3.51	0.70±2.01	1.26	3.99	Yes
$C_{n}^{6}$	−0.3±0.89	0.75	1.87	−0.25±0.68	0.56	1.42	Yes
$C_{n}^{7}$	−2.24±1.68	2.38	4.86	0.73±2.00	1.70	4.36	Yes
$C_{n}^{8}$	−0.37±1.36	1.11	2.79	0.17±0.95	0.75	1.91	Yes

### Discussion

This paper presents a graph-based automatic algorithm, OCTRIMA 3D, to segment cellular layers of the retina on macular scans from OCT volume data. The graph-based segmentation method solved the boundary detection problem by employing well-solved graph models, such as max-flow/min cut or shortest-path search and was found to be robust against the speckle noise and vessel disruption [17, 21]. In this work, shortest-path based graph search was used to detect eight boundaries in the macular region of OCT volume data. No training was needed in this work. Bias correction is not performed in our experiments as the systematic error is minimum in the dataset analyzed. Both accuracy and speed were evaluated in the comparison with manual labelings and two state of the art graph-based segmentation methods [17, 21]. The mean and standard deviation of the signed errors, the mean of the unsigned errors and the 95 percentile were reported to quantify the accuracy of OCTRIMA 3D. The detection errors of eight boundaries by OCTRIMA 3D were significantly lower (*p* < 0.001) than inter-observer difference in 100 Spectralis SD-OCT images from 10 subjects. The processing time for the whole OCT volume of 496 × 644 × 51 voxels (captured by Spectralis SD-OCT) is around 26 seconds and the average unsigned error is about 1 pixel. The Iowa reference algorithm is based on the minimum cost surface search on the graph constructed from 3D volume data. It is robust even when the boundary is missing in one of the frames due to vessel disruption or low signal strength. The detection result is smooth across the whole volume. However, the smoothness between frames could be a disadvantage in the following scenarios: (a) when there are motion artifacts in the OCT volume data and (b) when there are bumps or sudden curvature changes in the retinal structure. The first scenario is usually not found in the commercially available OCT devices due to the built-in motion correction algorithm. However, the algorithm may not work well in custom-built OCT devices without the implementation of motion correction algorithms. The smoothness constraint of the retina surface in the Iowa algorithm limited the capability of the algorithm to trace the small bumps and sudden curvature accurately. The Dufour’s software improves the Iowa Reference algorithm by adopting trained soft-constraint. The accuracy is greatly improved as illustrated in Fig 8 and Table 2.

The shortest-path based graph search method presented in [17] by Chiu et al. is the most related to our work. From the experiments, it was found that the processing speed of OCTRIMA 3D is greatly improved. The resulting improvement was mainly obtained as follows: 1. In Chiu et al.’s work, two graphs (dark-to-bright and bright-to-dark) were constructed from all the pixels in the OCT image for detecting eight boundaries. Most of the pixels were outside the search region resulting in a redundant process for the graph performance. In our methodology, a new graph for each boundary with a minimum number of vertices is constructed, facilitating a great reduction for the processing time; 2. Connectivity-based segmentation and several heuristic techniques were applied to define the search region for each boundary by Chiu et al. However, the heuristic techniques have limited robustness as images from different OCT modalities have different contrast and resolution. In OCTRIMA 3D, the connectivity-based segmentation was replaced with the masking and biasing operations; 3. The published work of Chiu et al. [17] did not consider the information from adjacent frames. In our implementation, the flattening and search region refinement step made use of inter-frame similarities.

Despite the promising results, our study has a few limitations. First, the processing time of the software is not only affected by the computational complexity, but also depends on the CPU processing power, programing language and number of tasks, which were not under control in the comparision between softwares. For example, the processing time of Chiu et al. algorithm is measured on a computer with differenct CPU power. The number of surfaces segmented by Iowa Reference software, Dufour’s software and OCTRIMA 3D were 11, 6 and 8, respectively. The programing languages used to deploy the algorithms were also different for the three software we compared. Therefore, the processing time of the softwares we measured is only a indicator of the real time capabilities. Second, the ground truth used the manual delineation of the retinal boundaries, which may be prone to inter-observer errors. Therefore, the manual labeling process was performed very carefully. On average, the labeling of eight boundaries on one OCT B-scan took 30 minutes to complete. Third, the current assumption is that the retinal surface is rather a flat surface and there are no big changes of boundary locations between frames (in the datasets analyzed/compared). This assumption works well for all of healthy cases. However, authors are aware that this assumption should be optimized in the presence of pathological features that may alter the retinal surface.

The most usual simplification approaches toward having real-time implementation require reducing both the number of operations and amount of data as well as the use of a simple or simplified algorithm. In this study, the processing time for the whole OCT volume has been greatly reduced while preserving the same amount of volume data.

### Conclusion and Future Work

In conclusion, a fast and accurate automatic segmentation algorithm, OCTRIMA 3D, has been developed to detect eight boundaries in macular scans from OCT volume data. OCTRIMA 3D methodology was developed based on the shortest-path graph search method proposed by Chiu et al. [17] and extended to 3D by making use of inter-frame similarities. The processing time for the whole volume was about 26 seconds and the average of unsigned detection error was about 1 pixel (about 4 microns). The processing time could be further improved by making use of parallel computing.

Overall, OCTRIMA 3D provides a fast, accurate and robust solution for the analysis of OCT volume data in real-time which could improve the usefulness of OCT devices in daily clinical routine. Future work will include the segmentation of peripapillary scans and corresponding tests of the algorithm robustness. As macular scans often seem more uniform than the peripapillary scans, we expect that some adjustments in OCTRIMA 3D may be needed. An evaluation of a larger OCT volume dataset of diseased eyes is also planned to investigate whether the method could be used as an aid to diagnose and monitor various retinal pathologies in a clinical setting.

## Supporting Information

S1 Video The video illustrates the use of OCTRIMA 3D software to segment Spectralis OCT 3D data. (MP4)

S1 Data The compressed folder contains the data from 10 subjects. For each subject, the *.mat file contains raw images used for segmentation, the results of OCTRIMA 3D, the manual labelings from Observer 1 and Observer 2.
(ZIP)
